# Two-year outcomes of UK patients newly diagnosed with atrial fibrillation: findings from the prospective observational cohort study GARFIELD-AF

**DOI:** 10.3399/BJGP.2021.0548

**Published:** 2022-05-17

**Authors:** Patricia N Apenteng, Saverio Virdone, FD Richard Hobbs, A John Camm, Keith AA Fox, Karen S Pieper, Gloria Kayani, David Fitzmaurice

**Affiliations:** Warwick Medical School, University of Warwick, Coventry.; Thrombosis Research Institute, London.; Nuffield professor of primary care health sciences, Nuffield Department of Primary Care Health Sciences, Radcliffe Primary Care Building, Radcliffe Observatory Quarter, University of Oxford, Oxford.; Cardiology Clinical Academic Group, Molecular & Clinical Sciences Research Institute, St George’s University of London, London.; Centre for Cardiovascular Science, University of Edinburgh, Edinburgh.; Thrombosis Research Institute, London.; Thrombosis Research Institute, London.; Warwick Medical School, University of Warwick, Coventry.

**Keywords:** all-cause mortality, anticoagulation, atrial fibrillation, bleeding, stroke

## Abstract

**Background:**

The outcomes of patients newly diagnosed with atrial fibrillation (AF) following the introduction of direct-acting oral anticoagulants are not well known.

**Aim:**

To determine the 2-year outcomes of patients newly diagnosed with AF, and the effectiveness of oral anticoagulants in everyday practice.

**Design and setting:**

This was a prospective observational cohort study in UK primary care.

**Method:**

In total, 3574 patients aged ≥18 years with a new AF diagnosis were enrolled. A propensity score was applied using an overlap weighting scheme to obtain unbiased estimates of the treatment effect of anticoagulation versus no anticoagulation on the occurrence of death, non-haemorrhagic stroke/systemic embolism, and major bleeding within 2 years of diagnosis.

**Results:**

Overall, 65.8% received anticoagulant therapy, 20.8% received an antiplatelet only, and 13.4% received neither. During the study period, the overall incidence rates of all-cause mortality, non-haemorrhagic stroke/systemic embolism, and major bleeding were 4.15 (95% confidence interval [CI] = 3.69 to 4.65), 1.45 (95% CI = 1.19 to 1.77), and 1.21 (95% CI = 0.97 to 1.50) per 100 person–years, respectively. Anticoagulation treatment compared with no anticoagulation treatment was associated with significantly lower all-cause mortality adjusted hazard ratio (aHR) 0.70 (95% CI = 0.53 to 0.93), significantly lower risk of non-haemorrhagic stroke/systemic embolism (aHR 0.39, 95% CI = 0.24 to 0.62), and a non-significant higher risk of major bleeding (aHR 1.31, 95% CI = 0.77 to 2.24).

**Conclusion:**

The data support a benefit of anticoagulation in reducing stroke and death, without an increased risk of a major bleed in patients with new-onset AF. Anticoagulation treatment in patients at high risk of stroke who are not receiving anticoagulation may further improve outcomes.

## INTRODUCTION

Atrial fibrillation (AF) increases the risk of ischaemic stroke fivefold and the risk of death twofold.^1^ AF-related strokes are more severe than strokes in people without AF and are more likely to be fatal, lead to long-term disability, extended hospital stays, and increased healthcare costs.^2^ Anticoagulation therapy reduces the risk of AF-related stroke (and systemic embolism) and death, with a 68% relative risk reduction for ischaemic stroke and a 25% reduction in the relative mortality.^3^ Anticoagulation, however, increases the risk of bleeding with the most serious complication being intracranial haemorrhage, which can be fatal.^4^

Anticoagulant drugs recommended by AF guidelines have included vitamin K antagonists (VKAs), usually warfarin, and direct-acting oral anticoagulants (DOACs), namely dabigatran, rivaroxaban, apixaban, and edoxaban.^4^^–^^6^ The latest National Institute for Health and Care Excellence (NICE) AF guideline (2021) recommends anticoagulation for patients with a CHA_2_D_2_-VASc score of ≥2 with a DOAC as a first-choice anticoagulant and VKA as an alternative if DOACs are contraindicated or not tolerated.^7^

Since the introduction of DOACs in clinical practice several UK studies have reported the clinical management of AF and changes in prescribing patterns, indicating increases in anticoagulant use overall and good uptake of DOACs.^8^^–^^12^ However, evidence on outcomes for UK patients newly diagnosed with AF at risk of stroke following the introduction of DOACs is limited.

This study investigates the 2-year event rates for non-haemorrhagic stroke/systemic embolism, all-cause mortality, and major bleeding in UK patients enrolled in the Global Anticoagulant in the FIELD — Atrial Fibrillation registry (GARFIELD-AF).

## METHOD

### Study design

GARFIELD-AF is a prospective, observational, international registry of adults (aged ≥18 years) newly diagnosed with AF.^13^ GARFIELD-AF was conducted in 35 countries worldwide, including the Americas, Europe, Africa, Asia-Pacific and the Middle East between 2010 and 2018. The UK study was conducted in accordance with the published study protocols.^13^^,^^14^ Participants were enrolled into five prospective sequential cohorts between 2011 and 2016. Inclusion criteria comprised males and females aged ≥18 years with a new diagnosis of non-valvular AF of up to 6 weeks before entry into the registry and an investigator-determined risk factor for stroke, meaning the risk factors for stroke were not pre-specified in the protocol and left to the clinical judgement of the site investigator.

**Table:** How this fits in

Atrial fibrillation (AF) increases the risk of stroke and death; anticoagulation reduces these risks at the cost of an increased risk of bleeding. There has been an increase in the proportion of patients with AF receiving anticoagulants, with patients receiving either vitamin K antagonists or direct-acting oral anticoagulants. Evidence on outcomes following the increase in the use of anticoagulant therapy in the UK is limited. In this study, the benefit of anticoagulation in this real-world cohort of patients with AF affirms recommendations in AF management guidelines. Addressing gaps in anticoagulation treatment for patients with AF may reduce AF-related stroke and death.

Eligible patients were recruited consecutively at participating sites to prevent selection bias. All participants provided informed consent. Patients were followed up for a minimum of 2 years (study end) or the occurrence of the event of interest, or loss to follow-up, whichever came first. Patients with transient AF, secondary to a reversible cause, and patients for whom follow-up was not possible were excluded.

### Setting

The UK specific study recruited from primary care, with 185 sites (GP practices) across the country. Participants were enrolled between June 2011 and August 2016. Data were collected from participants’ primary care records at baseline and at 4-month intervals up to 24 months post-diagnosis using an electronic case report file by trained local site staff.

### Variables

Data collected at baseline included patient characteristics, medical history, and antithrombotic therapy initiated at diagnosis. The main outcomes were non-haemorrhagic stroke and systemic embolism, major bleeding, and death. Major bleeding was defined as clinically overt bleeding associated with:

- a fall in haemoglobin of ≥2 g/dL; or
- a transfusion of ≥2 units of packed red blood cells or whole blood; or
- a critical site (intracranial, intraspinal, intraocular, pericardial, intra-articular, intramuscular with compartment syndrome, and retroperitoneal); or
- a fatal outcome.

The full classification of bleeding events can be found in Supplementary Box S1. Anticoagulant use was measured as anticoagulation prescribed at diagnosis. Anticoagulant use includes patients receiving anticoagulants with an antiplatelet (that is with or without an antiplatelet).

### Statistical analysis

Continuous variables are expressed as mean (standard deviation [SD]) and categorical variables as frequency and percentage. Use of antithrombotic therapy at baseline was analysed by CHA_2_DS_2_-VASc and HAS-BLED scores; HAS-BLED was modified to exclude fluctuations in the international normalised ratio as these data were not available. Females with no other risk factors were assigned a CHA_2_DS_2_-VASc score of 0. The occurrence of the major outcomes non-haemorrhagic stroke/systemic embolism, major bleeding, and mortality are presented using number of events and person–time event rate per 100 person–years and 95% confidence intervals (CIs). Only the first occurrence of each event was taken into account.

A propensity score was applied using an overlap weighting scheme to reduce biased estimates of the treatment effect. Weights were applied to Cox proportional hazards models to estimate the effects of the anticoagulant versus no anticoagulant comparison on the occurrence of death, non-haemorrhagic stroke/systemic embolism, and major bleeding within 2 years of enrolment. This newly developed method of overlap propensity weighting avoids excluding patients (as with matching) and gives the most weight to propensities where there is equipoise. This applied method overlaps weights and optimises the efficiency of comparisons by defining the population with the most overlap in the covariates between treatment groups. This scheme eliminates the potential for outlier weights by avoiding a weight based on a ratio calculation using values bounded by 0 and 1. Thus, when using overlap weights, many of the concerns regarding the assessment and the trimming of the weights are eliminated.^15^ Covariates evaluated in the weighting scheme included demographic characteristics (sex, age, and ethnicity), lifestyle factors (current smoking and alcohol consumption), clinical measurements at diagnosis (body mass index, heart rate, and blood pressure), medical history (congestive heart failure, acute coronary syndromes, vascular disease, carotid occlusive disease, prior stroke/transient ischaemic attack/systemic embolism, prior bleeding, venous thromboembolism, hypertension, hypercholesterolemia, diabetes, cirrhosis, moderate-to-severe chronic kidney disease, dementia, hyperthyroidism, and hypothyroidism), and baseline antiplatelet use.

Treatment was defined as the first treatment received at the time of enrolment, approximating ‘intention to treat’. Patients with missing values were not removed from the study; multiple imputation combining estimates from five imputed datasets was applied for the comparative effectiveness analysis.

Data analysis was performed centrally by study statisticians using SAS (version 9.4).

## RESULTS

### Participants

In total, 3574 patients were prospectively enrolled to the UK study, comprising 6.9% (*n* = 3574/52 057) of the global cohort. Of these, 2.5% (*n* = 89/3574) were lost to follow-up and had incomplete 2-year data. At baseline, the mean age was 74.5 (SD 9.5) years (data not shown), 42.6% (*n* = 1522/3574) of participants were female (Table 1), and 98.8% (*n* = 3441/3483) were White.

**Table 1.** Baseline characteristics of participants

**Variable**	**Value**	**%**
Age, years, median (Q1; Q3)	75.0 (69.0; 81.0)	—

**Age, years,** ***n/N***		
<65	471/3574	13.2
65–74	1178/3574	33.0
≥75	1925/3574	53.9

Sex, female, *n/N*	1522/3574	42.6

Ethnicity, White, *n/N*	3441/3483	98.8

Body mass index, median (Q1; Q3)	28.1 (25.0; 32.3)	—

**Medical history, *n/N***		
Congestive heart failure	274/3573	7.7
Coronary artery disease	678/3573	19.0
Acute coronary syndromes	363/3561	10.2
Carotid occlusive disease	52/3515	1.5
Prior stroke/TIA/systemic embolism	450/3551	12.7
Vascular disease^a^	760/3551	21.4
History of bleeding	109/3560	3.1
Hypertension	2483/3564	69.7
Hypercholesterolemia	1318/3492	37.7
Diabetes mellitus	629/3573	17.6
Chronic kidney disease (grade ≥3)	896/3499	25.6
Cirrhosis	11/3527	0.3

CHA_2_DS_2_-VASc, median (Q1; Q3)	3.0 (2.0; 4.0)	—

**CHA_2_DS_2_-VASc score categories, *n/N***		
0	89/3528	2.5
1	289/3528	8.2
2	659/3528	18.7
3	972/3528	27.6
4	848/3528	24.0
5	398/3528	11.3
≥6	273/3528	7.7

HAS-BLED score, median (Q1; Q3)^b^	2.0 (1.0; 2.0)	—

**HAS-BLED score categories,** ***n/N* ^b^**		
0	160/2530	6.3
1	941/2530	37.2
2	950/2530	37.5
3	391/2530	15.5
≥4	88/2530	3.5

**Care setting at diagnosis, *n/N***		
Cardiology	544/3573	15.2
Geriatrics	63/3573	1.8
Internal medicine	779/3573	21.8
Neurology	4/3573	0.1
Primary care/general practice	2183/3573	61.1

a *Defined as peripheral artery disease and/or coronary artery disease.*

b *The risk factor ‘Labile INRs’ is not included in the HAS-BLED score as these data are not collected at baseline. As a result, the maximum HAS-BLED score at baseline is eight points (not nine). INR = international normalisation ratio. Q1 = quartile 1. Q3 = quartile 3. TIA = transient ischaemic attack.*

The median CHA_2_DS_2_-VASc and HAS-BLED scores were 3.0 (quartile 1 [Q1] 2.0; quartile 3 [Q3] 4.0) and 2.0 (Q1 1.0; Q3 2.0), respectively (Table 1). In total, 89.3% (*n* = 3150/3528) had a CHA_2_DS_2_-VASc score of ≥2 and 18.9% (*n* = 479/2530) had a HAS-BLED score ≥3.

### Antithrombotic treatment

Of the participants, 65.8% (*n* = 2344/3564) received anticoagulant therapy at diagnosis; of these 70.6% (*n* = 1656/2344) received VKA and 29.4% (*n* = 688/2344) received a DOAC. In total, 12.5% (*n* = 447/3564) received anticoagulant therapy and an antiplatelet, 20.8% (*n* = 742/3564) received an antiplatelet only, and 13.4% (*n* = 478/3564) received neither anticoagulant nor antiplatelet therapy (data not shown).

Anticoagulant therapy was prescribed in 67.1% (*n* = 2108/3142) of patients with CHA_2_DS_2_-VASc score of ≥2, 56.1% (*n* = 161/287) of patients with CHA_2_DS_2_-VASc score of 1 (that is males with a score of 1) and 47.2% (*n* = 42/89) of patients with CHA_2_DS_2_-VASc score of 0 (that is females with a score of 1 and males with a score of 0). Of the participants, 51.6% (*n* = 247/479) with a HAS-BLED score ≥3 received anticoagulation (data not shown).

**Figure 1.**
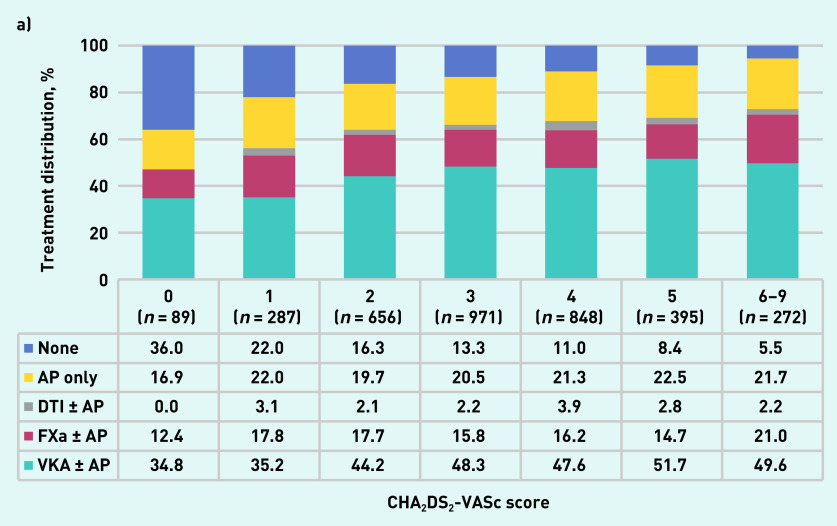

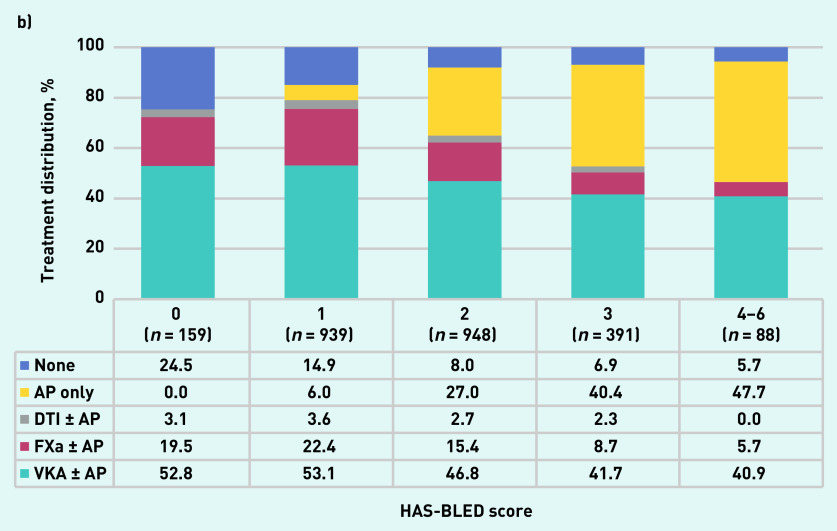
*a) Treatment at diagnosis by CHA_2_DS_2_-VASc score. b) Treatment at diagnosis by HAS-BLED score. AP = antiplatelet. DTI + AP = direct thrombin inhibitor and antiplatelet. FXa + AP = factor Xa inhibitor and antiplatelet. VKA + AP = vitamin K antagonist and antiplatelet.*

The proportion of patients receiving anticoagulant therapy increased progressively with CHA_2_DS_2_-VASc score (Figure 1a). The proportion of patients receiving anticoagulant therapy peaked in patients with a HAS-BLED score of 1, then decreased with increasing HAS-BLED score (Figure 1b). The proportion of patients receiving antiplatelet treatment only according to their CHA_2_DS_2_-VASc score ranged from 16.9% to 22.5% (Figure 1a). The proportion of patients receiving antiplatelet treatment only increased progressively with their HAS-BLED score from 0 in patients with a HAS-BLED score of 0 to 47.7% in patients with a HAS-BLED score from 4 to 6 (Figure 1b).

Most baseline characteristics were similar in patients who received anticoagulant therapy and patients who did not receive anticoagulant therapy; however, a higher proportion of patients receiving anticoagulation had hypertension, diabetes, prior stroke, and venous thromboembolism, whereas a higher proportion of patients with heavy alcohol consumption and history of bleeding did not receive anticoagulants (Table 2). This is of course due to hypertension, diabetes, and prior stroke being risk factors for AF-related stroke. Heavy alcohol consumption and history of bleeding are risk factors for bleeding.

**Table 2.** Baseline characteristics by anticoagulant treatment versus no anticoagulant treatment

**Baseline characteristics**	**Baseline treatment**		***P*-value^a^**

	**No OAC (*N*= 1219)**	**OAC (*N* = 2342)**	
**Sex, *n* (%)**			
Male	719 (59.0)	1324 (56.5)	0.161
Female	500 (41.0)	1018 (43.5)	

Age, years, median (Q1; Q3)	75.0 (69.0; 82.0)	75.0 (69.0; 81.0)	0.353

**Ethnicity, *n* (%)**	1172	2298	
White	1160 (99.0)	2268 (98.7)	0.659
Hispanic/Latino	2 (0.2)	8 (0.3)	
Asian	3 (0.3)	10 (0.4)	
African Caribbean/mixed/other	7 (0.6)	12 (0.5)	

Body mass index, kg/m^2^, median (Q1; Q3)	27.5 (24.6; 31.3)	28.4 (25.1; 32.7)	0.002

Systolic blood pressure, mmHg, median (Q1; Q3)	134.0 (121.0; 143.0)	132.0 (120.0; 140.0)	0.021

Diastolic blood pressure, mmHg, median (Q1; Q3)	79.0 (70.0; 84.0)	78.0 (70.0; 83.0)	0.127

Pulse, BPM, median (Q1; Q3)	82.0 (70.0; 102.0)	80.0 (70.0; 100.0)	0.033

**Type of atrial fibrillation, *n* (%)**			
Permanent	379 (31.1)	852 (36.4)	<0.001
Persistent	65 (5.3)	209 (8.9)	
Paroxysmal	257 (21.1)	392 (16.7)	
New onset (unclassified)	517 (42.4)	889 (38.0)	

**Medical history, *n* (%)^b^**			
Heart failure	70 (5.7)	201 (8.6)	0.003
Acute coronary syndromes	114 (9.4)	249 (10.7)	0.224
Vascular disease^c^	251 (20.7)	504 (21.7)	0.521
Carotid occlusive disease	14 (1.2)	37 (1.6)	0.294
Venous thromboembolism	40 (3.3)	124 (5.3)	0.007
Prior stroke/TIA/systemic embolism	122 (10.1)	328 (14.1)	<0.001
Prior bleeding	69 (5.7)	40 (1.7)	<0.001
Hypertension	810 (66.6)	1664 (71.3)	0.004
Hypercholesterolaemia	429 (36.1)	883 (38.5)	0.172
Diabetes	161 (13.2)	464 (19.8)	<0.001
Cirrhosis	6 (0.5)	5 (0.2)	0.155
Moderate-to-severe CKD	308 (25.9)	585 (25.5)	0.774
Dementia	8 (0.7)	20 (0.9)	0.529

Heavy alcohol consumption, *n* (%)^b^	68 (6.3)	58 (2.8)	<0.001

Current smoker, *n* (%)^b^	87 (7.3)	155 (6.7)	0.554

Antiplatelet treatment, *n* (%)	742 (60.9)	447 (19.1)	<0.001

CHA_2_DS_2_-VASc score, median (Q1; Q3)	3.0 (2.0; 4.0)	3.0 (2.0; 4.0)	<0.001

HAS-BLED score, median (Q1; Q3)	2.0 (1.0; 3.0)	2.0 (1.0; 2.0)	<0.001

a P*-values calculated using t-test or Wilcoxon-Mann-Whitney test for categorical variables and χ^2^ test or Fisher exact test for categorical variables, as appropriate.*

b *Some patients have unavailable baseline characteristics information. Percentages are calculated among those with available information.*

c *Defined as peripheral artery disease and/or coronary artery disease.*

d *The risk factor ‘Labile INRs’ is not included in the HAS-BLED score as it was not collected at baseline. As a result, the maximum HAS-BLED score at baseline is eight points (not nine). BPM = beats per minute. CKD = chronic kidney disease. INR = international normalisation ratio. OAC = oral anticoagulant. TIA = transient ischaemic attack.*

### Clinical outcomes

At 2-year follow-up, the incidence rates of all-cause mortality, non-haemorrhagic stroke/systemic embolism, and major bleeding were 4.15 (95% CI = 3.69 to 4.65), 1.45 (95% CI = 1.19 to 1.77), and 1.21 (95% CI = 0.97 to 1.50) per 100 person–years, respectively (data not shown).

**Figure 2.**
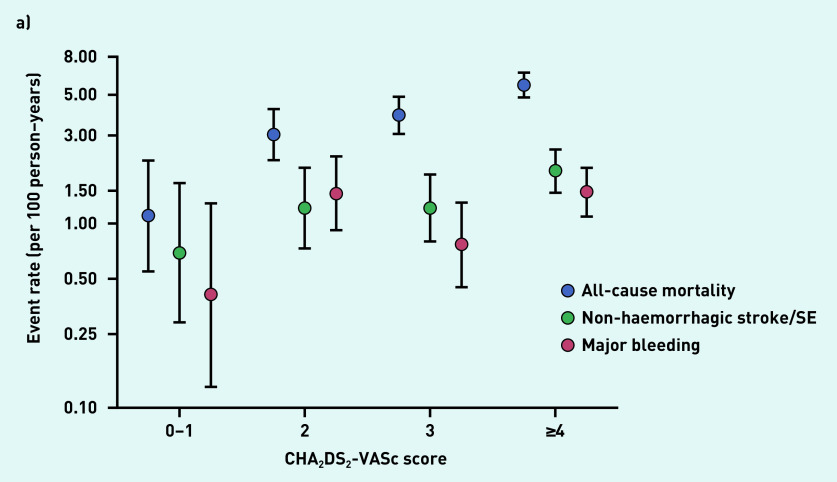

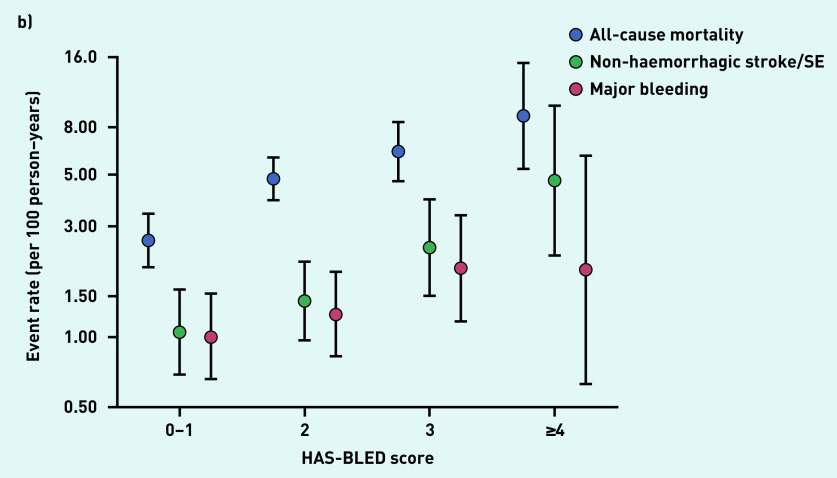
*a) Event rates according to CHA_2_DS_2_-VASc score. Includes only patients with available CHA_2_DS_2_-VASc scores (n = 3528). b) Event rates according to HAS-BLED scores. Includes only patients with available HAS-BLED scores (n = 2530). SE = systemic embolism.*

The rates of all-cause mortality, non-haemorrhagic stroke/systemic embolism, and major bleeding increased with increasing CHA_2_DS_2_-VASc and HAS-BLED scores (Figures 2a and 2b).

### Event rates by treatment at baseline

The incidence rates per 100 person–years of all-cause mortality, non-haemorrhagic stroke/systemic embolism, and major bleeding in patients who received anticoagulation were 3.89 (95% CI = 3.35 to 4.52), 1.05 (95% CI = 0.79 to 1.40), and 1.26 (95% CI = 0.97 to 1.64), respectively (Table 3). Comparatively, patients who did not receive anticoagulation had a higher rate of all-cause mortality and non-haemorrhagic stroke/systemic embolism (4.68, 95% CI = 3.87 to 5.66; 2.21, 95% CI = 1.67 to 2.92, respectively), and a lower rate of major bleeding (1.11, 95% CI = 0.75 to 1.65). Patients receiving DOAC had similar rates of all-cause mortality and non-haemorrhagic stroke/systemic embolism to patients receiving VKA (3.98, 95% CI = 3.03 to 5.23 and 1.00, 95% CI = 0.58 to 1.73 versus 3.85, 95% CI = 3.22 to 4.61 and 1.07, 95% CI = 0.76 to 1.51) but lower rates of major bleeding (0.77, 95% CI = 0.41 to 1.43 versus 1.46, 95% CI = 1.09 to 1.96).

**Table 3.** Two-year event rates per 100 person–years in the GARFIELD-AF UK population by treatment at baseline

**Treatment at baseline**	**All-cause mortality**		**Non-haemorrhagic stroke/systemic embolism**		**Major bleeding**	
	**Events**	**Rate (95% CI)**	**Events**	**Rate (95% CI)**	**Events**	**Rate (95% CI)**
OAC	172	3.89 (3.35 to 4.52)	46	1.05 (0.79 to 1.40)	55	1.26 (0.97 to 1.64)
No OAC	106	4.68 (3.87 to 5.66)	49	2.21 (1.67 to 2.92)	25	1.11 (0.75 to 1.65)
DOAC	52	3.98 (3.03 to 5.23)	13	1.00 (0.58 to 1.73)	10	0.77 (0.41 to 1.43)
VKA	120	3.85 (3.22 to 4.61)	33	1.07 (0.76 to 1.51)	45	1.46 (1.09 to 1.96)
OAC + antiplatelet	37	4.43 (3.21 to 6.11)	6	0.72 (0.32 to 1.61)	15	1.82 (1.10 to 3.02)
OAC only	135	3.77 (3.18 to 4.46)	40	1.13 (0.83 to 1.54)	40	1.13 (0.83 to 1.54)
Antiplatelet only	60	4.35 (3.37 to 5.60)	35	2.61 (1.87 to 3.63)	16	1.17 (0.72 to 1.91)
No OAC nor antiplatelet	46	5.19 (3.89 to 6.93)	14	1.60 (0.95 to 2.70)	9	1.02 (0.53 to 1.97)

*DOAC = direct-acting oral anticoagulant. GARFIELD-AF = Global Anticoagulant in the FIELD — Atrial Fibrillation registry. OAC = oral anticoagulant. VKA = vitamin K antagonist.*

The rates of all-cause mortality, non-haemorrhagic stroke/systemic embolism, and major bleeding in patients who received antiplatelet only were 4.35 (95% CI = 3.37 to 5.60), 2.61 (95% CI = 1.87 to 3.63), and 1.17 (95% CI = 0.72 to 1.91), respectively (Table 3).

### Effectiveness of anticoagulant use

**Figure 3.**
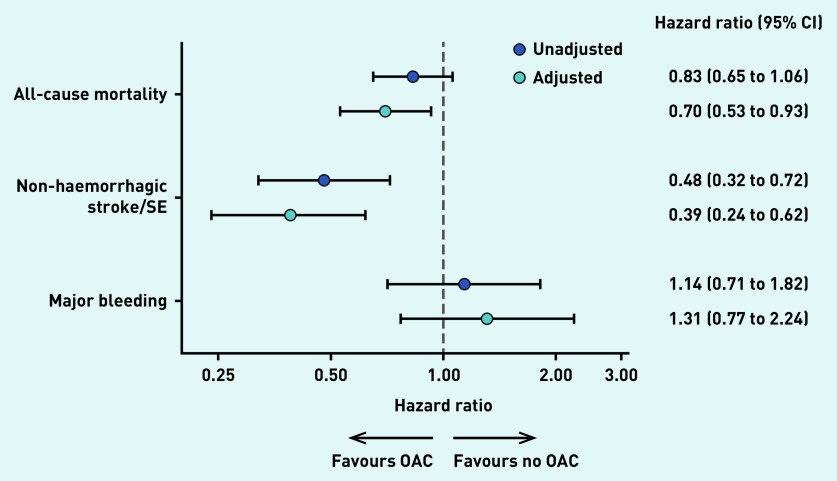
*Unadjusted and adjusted hazard ratios of OAC versus no OAC (reference) and corresponding 95% confidence intervals for selected outcomes at 2 years of follow-up in UK patients. Adjusted hazard ratios were obtained using an overlap-weighted Cox model. Variables included in the weighting scheme are: cohort enrolment, sex, age, ethnicity, type of AF, care setting specialty and location, congestive heart failure, acute coronary syndromes, vascular disease, carotid occlusive disease, prior stroke/transient ischaemic attack/SE, prior bleeding, venous thromboembolism, hypertension, hypercholesterolemia, diabetes, cirrhosis, moderate-to-severe chronic kidney disease, dementia, hyperthyroidism, hypothyroidism, current smoking, heavy alcohol consumption, body mass index, heart rate, systolic and diastolic blood pressure at diagnosis, and baseline antiplatelet use. AF = atrial fibrillation. OAC = oral anticoagulant. SE = systemic embolism.*

After adjustment for demographic and lifestyle factors (see Supplementary Figure S1), clinical measures at diagnosis, and medical history, anticoagulant use was associated with significantly lower all-cause mortality (adjusted hazard ratio [aHR] 0.70, 95% CI = 0.53 to 0.93, *P* = 0.013), non-haemorrhagic stroke/systemic embolism (aHR 0.39, 95% CI = 0.24 to 0.62, *P*<0.0001), and non-significant higher major bleeding (aHR 1.31, 95% CI = 0.77 to 2.24, *P* = 0.326) (Figure 3).

Treatment changes are described in Supplementary Box S2.

## DISCUSSION

### Summary

In this recent cohort of UK patients newly diagnosed with AF, death was the most frequent clinical outcome at 2 years occurring at 2.9 times the rate of non-haemorrhagic stroke/systemic embolism and 3.4 times the rate of major bleeding. Death remained the most frequent outcome regardless of whether patients were receiving anticoagulation or not. Anticoagulation treatment compared with no anticoagulation treatment was associated with significantly lower all-cause mortality, significantly lower risk of non-haemorrhagic stroke/systemic embolism, and a non-significant higher risk of major bleeding.

### Strengths and limitations

GARFIELD-AF was conducted to high-quality standards and data for 20% of the UK cohort were monitored against source documentation. Enrolling patients within 6 weeks of diagnosis ensured the sample included patients who may not survive long after an AF diagnosis by capturing disease burden early on.

The main limitation of the study is that the analysis is intention to treat, based on therapy initiated at diagnosis, and does not account for treatment changes during the 2-year follow-up. Also, the study did not collect data on deprivation and therefore it was not possible to adjust for deprivation in the analysis. Despite having applied appropriate propensity score methodology to balance confounding factors across treatment groups, the authors cannot exclude the presence of unobserved confounding.

### Comparison with existing literature

The findings of the present study regarding the benefit of anticoagulation fits with previous meta-analyses of randomised controlled trials in the VKA-only era as well as the DOAC trials.^16^^,^^17^^–^^20^ The estimated lower risk of 30% and 61% for all-cause mortality and non-haemorrhagic stroke/systemic embolism in UK patients on anticoagulants in this study are similar to the results from these studies. The estimated 31% higher risk of major bleeding did not reach statistical significance, which might be because of the relatively small number of these events in this cohort of patients.

As in the UK cohort, death was the most frequent outcome in the global cohort,^21^ occurring at over three times the rate of non-haemorrhagic stroke/systemic embolism and almost five times the rate of major bleeding. Nevertheless, the 2-year event rates per 100 person–years were numerically higher in the UK cohort compared with the global cohort excluding the UK: all-cause mortality 4.15 (95% CI = 3.69 to 4.65) versus 3.80 (95% CI = 3.68 to 3.93), non-haemorrhagic stroke/systemic embolism 1.45 (95% CI = 1.19 to 1.77) versus 0.97 (95% CI = 0.91 to 1.04), and major bleeding 1.21 (95% CI = 0.97 to 1.50) versus 0.96 (95% CI = 0.90 to 1.03). Overall, a similar proportion received anticoagulation at baseline (65.8% versus 66.9%); the median CHA_2_DS_2_-VASc score was similar for the UK and global cohort (median CHA_2_DS_2_-VASc 3.0 [Q1 2.0; Q3 4.0] versus 3.0 [Q1 2.0; Q3 4.0]) but the median HAS-BLED scores were higher in the UK cohort (median HAS-BLED 2.0 [Q1 1.0; Q3 2.0] versus 1.0 [Q1 1.0 to Q3 2.0]).

The reduction in the risk of all-cause mortality and non-haemorrhagic stroke/systemic embolism was more marked in the UK cohort compared with the global cohort (30% versus 18% and 61% versus 29%, respectively), and the increment in the risk of bleeding was not statistically significant in the UK but statistically significant in the global cohort (aHR 1.31 [95% CI = 0.77 to 2.24] versus 1.46 [95% CI = 1.1 to 1.86], respectively).^22^

Overall though, the findings on the relative effects of anticoagulation in the global study are reproduced in this UK-only cohort. Marginal differences might be because of the wider uncertainty around the obtained estimates in the UK data. In addition, the global analysis on effectiveness of anticoagulants was based on a different group of patients comprising patients enrolled to cohort 3 to 5 and patients with a CHA_2_DS_2_-VASc score of ≥2.

### Implications for practice

Findings regarding the benefit of anticoagulation for reduction of all-cause mortality and non-haemorrhagic stroke/systemic embolism without a significant increase in the risk of bleeding suggests that for most patients the benefits of anticoagulation outweigh the risks, and affirms recommendations relating to anticoagulation in AF management guidelines.

The findings regarding a non-significant increase in the risk of bleeding are reassuring, particularly as 47.0% of patients with a HAS-BLED score of 4–6 received anticoagulation. Nevertheless, this finding should be interpreted with caution, and it must be emphasised that despite the benefit, anticoagulation is recommended for patients at increased risk of stroke with a CHA_2_D_2_-VASc score of ≥2 and should be considered for those patients with a CHA_2_D_2_VASc score of 1.

There is scope for improvement in the management of patients newly diagnosed with AF to align with NICE guidelines.^7^ Overall, 67.1% of participants at high risk of stroke (CHA_2_D_2_-VASc score ≥2) received anticoagulation at diagnosis. The authors have previously reported a progressive increase in the proportion of patients in the UK cohort receiving anticoagulation, with 75.6% of the final cohort of patients (diagnosed June 2015 to July 2016) receiving anticoagulation.^23^ Further increment in the proportion of patients at high risk of stroke receiving anticoagulation will optimise anticoagulation in patients with AF and improve outcomes. On the other hand, 47.2% of patients defined as very low risk in the NICE guidelines (CHA_2_D_2_-VASc score of 0 for males or 1 for females) received anticoagulation, which is contrary to the guidelines.

In addition, the practice of prescribing antiplatelet treatment alone (20.8%) is contrary to AF guidelines; the guidelines indicate patients at risk of stroke must receive anticoagulants or no antithrombotic therapy. The practice of prescribing anticoagulant with antiplatelet treatment (12.5%) is also not recommended in the guidelines.

The older profile of the patient population and the prevalence of comorbidities are likely to be contributory factors to the all-cause mortality. The prominence of all-cause mortality as an outcome indicates more attention should be given to mortality risk in the management of patients newly diagnosed with AF.

## Data

Requests for patient-level data can be made to the head of statistics at the Thrombosis Research Institute (Karen S Pieper, kpieper@tri-london.ac.uk). These requests should include a protocol summary and a summary of the statistical analysis plan. The request will be reviewed by the data-sharing committee for approval and next steps will be discussed with the requestor.
